# Wolves Are Better Imitators of Conspecifics than Dogs

**DOI:** 10.1371/journal.pone.0086559

**Published:** 2014-01-29

**Authors:** Friederike Range, Zsófia Virányi

**Affiliations:** 1 Messerli Research Institute, University of Veterinary Medicine Vienna, Medical University of Vienna, University of Vienna, Vienna, Austria; 2 Wolf Science Center, Ernstbrunn, Austria; CNR, Italy

## Abstract

Domestication is thought to have influenced the cognitive abilities of dogs underlying their communication with humans, but little is known about its effect on their interactions with conspecifics. Since domestication hypotheses offer limited predictions in regard to wolf-wolf compared to dog-dog interactions, we extend the cooperative breeding hypothesis suggesting that the dependency of wolves on close cooperation with conspecifics, including breeding but also territory defense and hunting, has created selection pressures on motivational and cognitive processes enhancing their propensity to pay close attention to conspecifics’ actions. During domestication, dogs’ dependency on conspecifics has been relaxed, leading to reduced motivational and cognitive abilities to interact with conspecifics. Here we show that 6-month-old wolves outperform same aged dogs in a two-action-imitation task following a conspecific demonstration. While the wolves readily opened the apparatus after a demonstration, the dogs failed to solve the problem. This difference could not be explained by differential motivation, better physical insight of wolves, differential developmental pathways of wolves and dogs or a higher dependency of dogs from humans. Our results are best explained by the hypothesis that higher cooperativeness may come together with a higher propensity to pay close attention to detailed actions of others and offer an alternative perspective to domestication by emphasizing the cooperativeness of wolves as a potential source of dog-human cooperation.

## Introduction

Based on comparing dogs and wolves in two communicative tasks with humans (pointing [1], [2], [3], [4] and ‘asking for help’ [5]), a range of hypotheses have been put forward to explain whether and how dog cognition might have changed during the course of domestication [1], [5], [6], [7]. While most of these hypotheses agree that dogs should perform better in animal-human cooperative interactions, they offer limited and different suggestions on how cognitive abilities in wolves and dogs would influence interactions with conspecifics. On one side of the continuum, the ‘information processing hypothesis’ by Frank [8] predicts that - due to the buffering effect of humans leading to a relaxation of natural selection on the problem-solving abilities of dogs - wolves perform better than dogs in cognitive tasks that rely on causal understanding and insight but it offers no prediction in regard to wolves’ and dogs’ social interactions with conspecifics. The ‘emotional reactivity hypothesis’ in its earlier form [6] suggested that during domestication dogs have become able to use their within-species cognition with humans and seems to imply that the intraspecific social skills of dogs and wolves are similar. More recently, however, it has been claimed that dogs evolved increased tolerance compared to wolves in general and are less aggressive also with conspecifics [9]. In the latter case, the emotional reactivity hypotheses would predict that dogs outperform wolves at least in tasks were closeness with conspecifics is of advantage (e.g. cooperation and social learning tasks). Finally, the ‘direct selection hypothesis’ [1] and the synergestic hypothesis [3] both predict dogs to outperform wolves due to their more human-like cognitive skills or to their better action inhibition respectively in comparison to wolves. A broader comparative perspective, focusing on social interactions with conspecifics rather than just association with humans is likely to provide additional insight into dog and wolf cognition. The social life of dogs and wolves strongly differ, not only in terms of human influence but also in intraspecific contexts.

Wolves are cooperative breeders where non-breeding pack members help the dominant pair(s) to raise their young [10], [11] and, moreover, they rely on close action-coordination with pack members when defending their territories and hunting large game [10], [11]. Dogs, however, though phylogenetically so closely related to the wolves that they often are considered to be the same species [12], [13], [14], differ fundamentally not just in regard to their closeness to humans, but also in their breeding system and possibly other intraspecific interactions ([15], [16]; but see [17]). Although feral dogs live in pack-like social groups and display differentiated social relationships with each other [18], [19], female feral dogs raise their pups alone [15], [20] or with the help of the fathers that in some populations may contribute to the defence of the pups but rarely feed them [21]. The cooperative breeding hypothesis [22], [23], [24] proposes that the motivational and cognitive processes required for behavioral coordination and helping in cooperative breeding, such as attentional biases toward monitoring others, the ability to coordinate actions spatially and temporally, pro-social attitudes and high social tolerance as well as responsiveness to others’ signals, have favored similar changes also in other contexts not directly related to care-giving, such as learning from observing others. Consequently, the overall higher dependency on cooperative interactions with conspecifics that require close action-coordination in order to be successful, predicts that wolves show a higher propensity to pay close attention especially to the actions of conspecifics and consequently can better learn from them.

Social learning is likely to be relevant for group-living mammals such as social carnivores [25], and paying close attention to others is an important prerequisite [26]. To date social learning has not been investigated in wolves, making it impossible to compare them with dogs that have been shown to readily learn from observing a conspecific model in several studies [27], [28]. Moreover, in regard to imitation, even in dogs the results are mixed. For example, Tennie et al. [29] found no evidence that dogs would copy the intransitive movements (sit and lie) of another dog. So far, only one study found that dogs are capable of imitating a conspecific in using either their paw or mouth to manipulate an apparatus in order to get food [30]. In general it seems that while dogs are able to socially learn and even imitate each other, this ability may be restricted to specific situations and/or may require specific training of the dog. Currently, in lack of knowledge on wolves, this data can be seen in contradictory ways. It is questionable whether this limited success of dogs to imitate conspecifics should be viewed as a by-product of human-induced improvement of dog skills to learn socially from humans or as a reminiscent of advanced imitation expected in the more cooperative wolves. To test this latter prediction of the social *canine intraspecific cooperation hypothesis*, an extension of the cooperative breeding hypothesis, we investigated the performance of wolves and dogs in a social learning task, involving conspecifics. Albeit the demonstrators for both dogs and wolves were trained pet dogs, we use the term conspecific in case of both groups in order to contrast our study with former studies that investigated the effects of domestication on the interactions of dogs and wolves with humans. From a systematic perspective, wolves and dogs are often categorized either as 2 species, 2 subspecies or 2 forms - domesticated and wild - of the same species. Either choice would probably provide an oversimplified static picture; dogs and wolves are rather at the beginning of the evolutionary process of a potential speciation. It is reflected in the fact that although their living environment and to some extent their behavior differ, dogs and wolves are able to form mixed groups in which they establish social relationships [31], but they can also interact with each other even without being socialized with members of the other “species” (e.g. can mate and produce offspring together) [32], [33].

Accordingly, we tested how the demonstration of a familiar dog influenced the performance of 6-months old wolves (n = 14) and mongrel dogs (n = 15) in a social learning task using a two-action imitation test. The wolves and the dogs were hand raised and kept in packs in identical conditions at the Wolf Science Center, ensuring that they had similar experiences and similar relationship with the conspecific models of this study. The dogs (N = 13) were retested approx. 9 months after the initial study to verify that dogs’ delayed development did not drive the observed difference between the wolves and dogs. Finally, 5 wolves, 6-months old or older, participated in a control condition to investigate whether wolves can solve the same problem also without demonstration.

## Methods

### Ethical Statement

No special permission for use of animals (wolves) in such socio- cognitive studies is required in Austria (Tierversuchsgesetz 2012– TVG 2012). The relevant committee that allows running research without special permissions regarding animals is: Tierversuchskommission am Bundesministerium für Wissenschaft und Forschung (Austria). The owners of the pet dogs that were present during the hand raising and part of this study (see below) gave permission for their animals to be used in this study.

The Wolf Science Center is located in the game park Ernstbrunn (License No.: AT00012014). The Cites permits for our animals are: 2008: Zoo Herberstein, Austria: AT08-B-0998, AT08-B-0996, AT08-B-0997; 2009: Zoo Basel, Switzerland: AT09-E-0061, Triple D Farm, USA: AT09-E-0018; 2010: Parc Safari, Canada: AT10-E-0018; 2012: Minnesota Wildlife Connection, USA: 12AT330200INEGCJ93, Haliburton Forest, Canada: AT12-E0020.

Two of the control wolves were located in the Gamepark Lüneburgerheide (License No.: DE 00016905). The Cites permits for the animals are: 2010: Parc Safari, Canada: AT10-B-1219; 2001: Lüneburger Heide, Germany: 1226/2001.

### Subjects

We tested 16 6-months-old wolves and 15 6-month-old mixed-breed dogs that had been raised and kept identically at the Wolf Science Center. Additionally, we tested 2 adult wolves (>2 years) hand-raised by Tanja Askani and kept at the Gamepark Lüneburger Heide in Germany. Moreover, all but 2 dogs were retested at adult age (>1.5 year) using exactly the same methods as when tested previously.

All wolves originated from North America and were born in captivity, while all dogs were obtained from animal shelters in Hungary. Please see table 1 for details on the sex, relatedness and year of birth of all animals. All animals (dogs and wolves separately at different times) were hand-raised with conspecifics in peer groups after being separated from their mothers in the first 10 days after birth. They were bottle-fed and later hand-fed by humans and had continuous access to humans in the first 5 months of their life. During the first weeks of puppyhood, the animals were kept inside, but from their second month on, they had free access to a 1000 m^2^ outside enclosure.

**Table 1:** List of animals, indicating genetic relationships (litter), sex (**M**ale/**F**emale), age, origin and type of demonstration received.

	Name	Sex	Litter	Born	Breeding facility	Demonstration
Wolf	Aragorn	M	1	2008	Herberstein, Austria	Paw*
Wolf	Shima	F	1	2008	Herberstein, Austria	Mouth*
Wolf	Kaspar	M	2	2008	Herberstein, Austria	Mouth+
Wolf	Tatonga	F	4	2009	Tripple D Farm, USA	Paw
Wolf	Nanuk	M	3	2009	Tripple D Farm, USA	Mouth
Wolf	Geronimo	M	5	2009	Tripple D Farm, USA	Paw
Wolf	Yukon	F	5	2009	Tripple D Farm, USA	Mouth
Wolf	Cherokee	M	6	2009	Zoo Basel	Paw
Wolf	Apache	M	6	2009	Zoo Basel	Mouth
Wolf	Kenai	M	7	2010	Parc Safari, Canada	Paw
Wolf	Wapi	M	7	2010	Parc Safari, Canada	Mouth
Wolf	Tala	F	8	2012	Minnesota Wildlife Connection, USA	Paw
Wolf	Amarok	M	8	2012	Minnesota Wildlife Connection, USA	Control
Wolf	Una	F	9	2012	Minnesota Wildlife Connection, USA	Control
Wolf	Chitto	M	9	2012	Minnesota Wildlife Connection, USA	Paw
Wolf	Wamblee	M	10	2012	Haliburton Forest, Canada	Control+Paw
Wolf	Naaja	F	7	2010	Parc Safari, Canada	Control
Wolf	Nanuk	M	12	2001	Wildpark Lündeburger Heide	Control
Dog	Rafiki	M	1	2009	Tengelic; Hungary	Paw
Dog	Alika	F	1	2009	Tengelic; Hungary	Mouth
Dog	Kilio	M	2	2009	Paks, Hungary	Mouth
Dog	Maisha	M	2	2009	Paks, Hungary	Paw
Dog	Asali	M	3	2010	Siofok, Hungary	Mouth
Dog	Binti	F	3	2010	Siofok, Hungary	Paw
Dog	Bashira	F	4	2010	Paks, Hungary	Mouth
Dog	Hakima	M	4	2010	Paks, Hungary	Paw
Dog	Meru	M	5	2010	Velence, Hungary	Mouth
Dog	Nuru	M	6	2011	Paks, Hungary	Paw
Dog	Zuri	F	6	2011	Paks, Hungary	Mouth
Dog	Layla	F	7	2011	Györ, Hungary	Paw
Dog	Bora	F	7	2011	Györ, Hungary	Mouth
Dog	Nia	F	8	2011	Paks, Hungary	Mouth
Dog	Yera	M	9	2011	Paks, Hungary	Mouth

* Excluded from analyses due to neophobic reactions;

+ Was neophobic first, but then readily approached the box on the second day. Data were not included for latency calculations in the analyses.

At five months they were moved to a 2000–8000 m^2^ enclosures, where there were no humans continuously present, but all animals participated in training and/or cognitive and behavioural experiments at least once a day and hence had social contact with humans. This daily routine assures that both, wolves and dogs, are cooperative and attentive towards humans and also allows veterinary checks without sedating the animals. Also, five adult pet dogs of various breeds had regular (2–3 days per week) but not continuous contact with the wolves and dogs during the first 6 months of their lives. They established close relationships with the wolves and dogs (greeting them, playing with them, establishing dominance relationships) and until the end of this study all wolves and dogs readily submitted to these pet dogs, two of which were used as demonstrators for the current study (see below). Thus, both wolves and dogs had the same extensive experience with conspecifics throughout their first 6 months of life as well as the same experience with the pet dogs used as demonstrators in this study (see below).

The puppy enclosure as well as the later living enclosures were equipped with trees, bushes, logs and shelters. Water for drinking was permanently available. The wolves had a diet of meat, fruits, milk products and dry food throughout the study period. During the first months of their lives, they were fed several times per day, which was slowly reduced to being fed major meals twice or three times per week according to their natural rhythm. The dogs received a similar diet but received smaller portions of food every day.

A testing room (6×10 m) next to the enclosures allowed for training and testing the animals in isolation from the pack. The testing room was empty except of a table in the first 3 wolf- and first 2 dog generations. It was not possible to look outside through the windows in the testing room. All animals worked in separation from their pack members on a daily basis. Participation in all training and testing sessions was voluntary.

The two American wolves handraised by Tanja Askani grew up in her private house. Although these animals were not raised with peers, they had regular contact with a pack of adult wolves as well as with dogs throughout the raising period. They were introduced to the pack at the age of 3 to 4 months. Although these wolves do not have the same training regime as the wolves at the Wolf Science Center, they are very well socialized with strangers as well as with unfamiliar dogs, are regularly visited in the enclosures, receive various kinds of enrichment and are taken out for walks on the leash until they are 3 to 4 years old.

### Experimental Set-up

**Figure 1:**
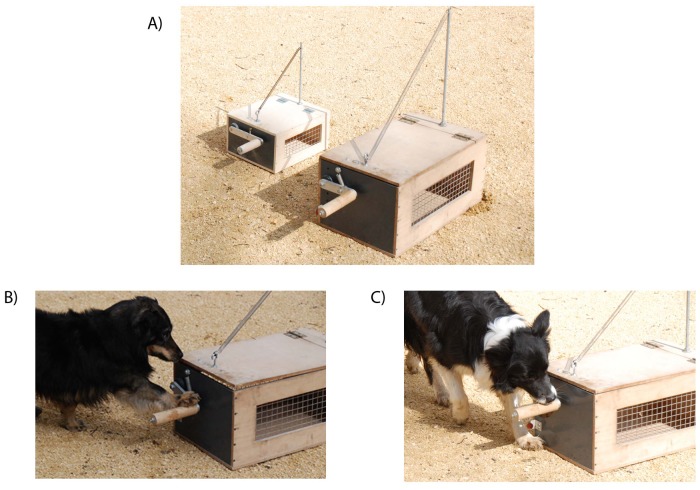
Pictures of the test apparatus and the two kinds of demonstrations. A) the two different sizes of the experimental apparatuses; B) a paw demonstration; C) a mouth demonstration.

The animals were presented with a novel instrumental problem-solving task, where they had to push down a wooden lever to open the lid of a box to access a food reward hidden in the box (Figure 1a,b). All of the dogs and 14 of the wolves were presented with repeated demonstrations (see below) by one of two familiar dogs dominant over the subjects opening the box using either its paw (Figure 1a) or its mouth (Figure 1b). In a subsequent test, the subjects were released to manipulate the baited apparatus to see if and how they solve the task and whether they match their behavior to the demonstrated action. In a control group, the wolves (n = 5, including the 2 adult wolves) were subjected to the same procedure as the test animals, but the opening of the box itself was performed behind a screen and thus invisible to the subjects. One of the control animals at the Wolf Science Center, Wamblee, that was unsuccessful in the control trial, was retested watching a full demonstration. Since Wamblee had tried to manipulate the box with its mouth in the control trial, it was subjected to a paw demonstration (e.g. in order to copy the demonstrated action, it would have had to change its preference). If however, the analyses differed according to whether Wamblee was included or not, we present the statistical results without Wamblee in parentheses.

All but three subjects were tested in the testing room of the Wolf Science Center. The subject, three experimenters (E1, E2, E3) and a dog as demonstrator were present during the experiments. The two adult wolves participating in the control condition were tested in their living enclosure at the Game park Lüneburger Heide, after separating the other animals in an airlock system with different compartments. While from the first compartment it was possible to observe the experiment, from the other compartments one could not observe what was happening in the living enclosure.

### Test Apparatus

The apparatus used for the experiment was a rectangular wooden box covered by a lid that opened if a lever on the front side of the box was pushed down with either mouth or paw (see Figure 1 c). During testing the box was filled with heavy stones so that it would not move easily. Either a bigger or a smaller version of this box was used, matched to the size of each animal (length * width * height: small box = 32×26×16 cm; large box = 52×32×25 cm) to avoid that potential differences between wolves and dogs could be attributed to physical differences. The sides of the boxes consisted of wire mesh, while the front, the rear part and the lid were made out of solid wood.

### Procedure

#### Training of the demonstrator dogs

The demonstrator dogs were trained using a shaping procedure with a secondary reinforcer (clicker) to open the box with the required method (paw/mouth). Both dogs learned how to do the demonstration reliable within a few training sessions each consisting of less than 10 trials. Their performance was checked again conducting 3–5 training trials before each experiment.

#### Demonstration phase

While experimenter 2 (E2) was holding the subject on the leash, she turned it away from the box occupying the animal by asking for simple commands that the animals are trained on (like sit, look) so that it could not see the box. During this procedure, experimenter 1 (E1) baited the box. To avoid that the animals could by any chance see when E1 manipulated the box, she baited the box while positioning herself between the box and the position where E2 was occupying the animal, and closed the lid. After baiting, E1 returned to her starting position, and the subject was turned to watch the demonstration. Once the subject was looking into the direction of the specific demonstrator dog, E1 sent the model dog to open the box with the command ‘box’. E2 did not talk to the subject during the demonstration. After the subject had seen the demonstration and the dog demonstrator had returned to E1, E2 led the subject to the open box where it could retrieve the treat from inside the box. If needed in the first trial, E2 pointed to the food and encouraged the subject to take it. The subject was not allowed to inspect the box but merely was permitted to take the treat before being led away by E2. The whole procedure was repeated immediately until the subject had witnessed a total of six demonstrations. If the subject did not pay attention to one of the demonstrations, additional demonstrations were conducted so that each animal observed a total of six demonstrations (additional demonstrations: 7 dogs and 1 wolf:+one; 3 wolves:+two; 1 dog:+three). Neither the demonstration nor the test trials were accompanied by communicative cues or encouragement by the experimenters except in the first demonstration trial if the subject was hesitating to take the food (see above). Please see video S1 and S2 for an illustration of the procedure.

In each demonstration the subject saw the same model using the same method to open the box (either with the paw or with the mouth). Subjects were pseudo-randomly assigned to the two kinds of demonstration so that sex and relatedness were counterbalanced.

#### Test phase

Right after the last demonstration, E2 again turned the subject away from the box, while E1 baited it. E3 started to record the action of the subject with a portable camera. E2 then turned the subject around and started the trial by unleashing the subject. All experimenters remained at their positions and did not encourage the subject in any way. If the subject managed to open the box it was allowed to eat the food, and the trial was terminated. Otherwise the trial was terminated after 5 minutes. If the animal was successful, the subject was taken back into the starting position, and further trials followed - up to 6 trials per animal (please see video S3 and S4 for an illustration of the first test trials).

Since three wolves showed clear neophobic reactions towards the box during the 6 demonstration trials and did not approach the box in the first test trial, we familiarized these animals by providing food in the open box until they took the food relaxed (they were not allowed to manipulate the box at any time during the familiarization; see table 1 for identities of these wolves). One day later, the three animals received two more demonstrations and then were tested again. While two of the three wolves were still afraid and thus were excluded from the analyses, one male now readily approached and manipulated the apparatus. However, due to the extra habituation, we did not include the latencies in the analyses. None of the other wolves nor dogs showed any neophobic reactions towards the box (i.e. all manipulated the box in the first test trial).

#### Control trials

The control experiment at the Wolf Science Center was conducted in exactly the same way as the demonstration and test phase described above with the only difference that during the demonstration, a partition (80 cm×120 cm) was used to render the demonstrator dog opening the box invisible.

At the Game park Lüneburger Heide the ‘demonstrator’ dog was unfamiliar to the wolves, thus each subject watched the occluded demonstration from a compartment of the airlock system, and the dog was taken out of the enclosure before the wolf entered e.g. in between the demonstration trials when the wolf could retrieve the food from the box and during the test phases. However, none of the wolves showed any aggression towards the dog, but instead were highly interested in her. Nevertheless, they immediately approached the box and searched for the food as soon as released into the enclosure.

### Analysis

From the test videos, we extracted the latency to approach the box to within 1 meter, the latency to investigate (sniffing) and manipulate (scratching, chewing, pushing with the nose) the different parts of the box (relevant for opening: lever, front; non-relevant for opening: side, back) in the first trial the animal manipulated the box as well as the success (whether or not the subject managed to open the box). Moreover, we coded which method the subject used to manipulate the box (mouth or paw) the first time as well as which method it succeeded with in opening the box. Accidental opening, i.e. when the animals ran against the lever or manipulated the side/back of the box so hard that it opened, were disregarded. Finally we coded the latencies when the animals look at the face of one of the present experimenters for the first time. The coding of the test videos started when the subject was released from the leash by the experimenter and ended once the trial was terminated. Time resolution was set at 0.20 seconds.

Test videos were analyzed by a coder who was blind to the experimental conditions. To confirm scoring consistency another independent person coded 30% of the videos. Spearman rank correlations were perfect for most variables (rho = 1), and very good for approaching the box within 1 m (rho = 0.85) and the first manipulation of the front part of the box (rho = 0.91).

## Results

### Performance of Wolves and Dogs in the First Test Trial at 6 Months of Age

The wolves and dogs did not differ from each other in the latency from being released to approaching the apparatus in their first trial (exact Mann-Whitney U test, N_W_ = 10, N_D_ = 15, z = −0.77, p = 0.45). More interestingly, they also did not differ in their latency to investigate the most relevant part of the box, the lever (exact Mann-Whitney U test, N_W_ = 9, N_D_ = 15, z = −0.12, p = 0.92; one wolf was excluded since it was not visible on the respective video record when and where she investigated the box for the first time) or in the latency to manipulate the front of the box (including the lever) (exact Mann-Whitney U test, N_W_ = 10, N_D_ = 15, z = 1.42, p = 0.16). The latency data of Wamblee (the control wolf that was retested with a demonstration) were not included in these analyses, but data on which part and how (paw/mouth) this animal manipulated the box as well as its success were included in the following analyses.

**Figure 2:**
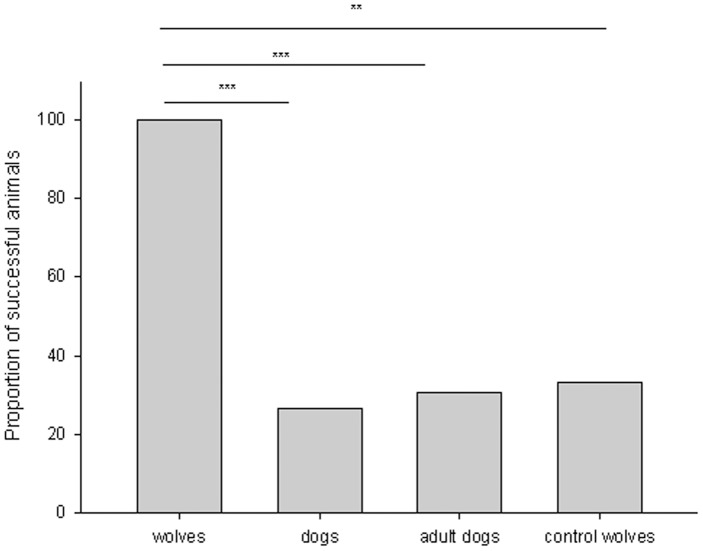
The graph depicts the proportion of animals that successfully opened the box in their first trials in the 4 test groups (wolf and dog test groups, adult dog control group, wolf control group).

When investigating which part of the box the wolves and dogs manipulated first, we only found a tendency for a difference (Fisher exact test: p = 0.087; without Wamblee: p = 0.038). Dogs were more likely to manipulate first another part of the box rather than the lever (binomial test, testing for 50% probability here and later: p = 0.007; 2 handle vs. 13 other parts of the box), whereas no differences was found in the wolves (binomial: p>0.99; 6 handle vs. 6 other parts). Finally, wolves and dogs differed in regard to their success (Fisher exact test: p<0.001) with all wolves opening the box at least once by actively manipulating the lever (binomial test: p<0.001; N = 12), while only 4 dogs out of the 15 were successful (binomial test: p = 0.12, see figure 2).

Importantly, the difference in success between the wolves and the dogs in the first trial is unlikely to be due to the dogs trying to use first a different strategy such as asking for help from a human (see [5]): First, the dogs and wolves actually did not differ in their first interactions with the boxes (see above), showing that their first attempt was to manipulate the box themselves. Second, the wolves manipulated the box significantly earlier than the unsuccessful dogs looked at any of the humans present during the test (exact Mann-Whitney U test: N_W_ = 12, N_D_ = 11, z = −3.873, p<0.001). Finally, there was no significant difference in the wolves’ latency to success and the unsuccessful dogs’ latency to look at a human (Mann-Whitney-U: N_W_ = 12, N_D_ = 11, z = −0.90, p = 0.39).

Regarding imitation, we investigated first whether wolves and dogs would use the same methods as demonstrated when they manipulated the box for the first time. We found that the wolves significantly matched the demonstrated action (binomial test: p = 0.040; 10 same vs. 2 different; without Wamblee: p = 0.065; 9 same vs. 2 different), whereas dogs used two 2 methods more randomly (binomial test: p = 0.607; 9 same vs. 6 different). While based on this variable action-matching did not differ between wolves and dogs (Fisher exact test: p = 0.24), they differed significantly from each other in regard to whether or not they matched the demonstrated action during their successful manipulation (Fisher exact test: p = 0.019). None of the four successful dogs used the same method as demonstrated to open the box (binomial test: p = 0.125). Though 9 of the 12 wolves (without Wamblee: 8 out of 11 wolves) matched the observed method to successfully manipulate the lever, this proportion is non-significant (binomial test: p = 0.15).

### Performance of Wolves and Dogs in the Test Trials 2–5 at 6 Months of Age

Eight of the 12 wolves continued to successfully open the box across the 6 trials, one animal stopped after the third opening, one after the second opening and 2 refused to further manipulate the box after their first success (one because he got scared when the box opened). In contrast, only two of the 4 successful dogs continued to open the box. The difference between the number of wolves and dogs that succeeded in more test trials was not significant (Fisher exact test: p = 0.60, see table 2 for successes of animals across trials).

**Table 2:** Success of animals in the various trials.

	Name	Trial 1	Trial 2	Trial 3	Trial 4	Trial 5	Trial 6
Wolf	Apache	AC	AC	1	1	1	1
Wolf	Cherokee	1	1	1	1	1	1
Wolf	Chitto	1	1	0			
Wolf	Geronimo	1	0				
Wolf	Kaspar	1	1	1	1	1	1
Wolf	Kenai	1	1	1	1	1	1
Wolf	Nanuk	1	1	1	1	1	NA
Wolf	Tala	1	1	1	0		
Wolf	Tatonga	1	1	1	1	1	1
Wolf	Wamblee	1	0				
Wolf	Wapi	1	1	1	1	1	NA
Wolf	Yukon	1	AC	AC	AC	AC	AC
Dog	Alika	0					
Dog	Asali	0					
Dog	Bashira	0					
Dog	Bora	0					
Dog	Binti	1	0				
Dog	Hakima	0					
Dog	Kilio	0					
Dog	Layla	0					
Dog	Maisha	0					
Dog	Meru	1	0				
Dog	Nia	1	1	1	1	0	
Dog	Nuru	0					
Dog	Rafiki	0					
Dog	Yera	0					
Dog	Zuri	1	1	1	1	1	0

If animals were not successful in a given trial, they were not tested further.

AC = Accidental opening, NA = Not available.

### The Influence of Development of Dogs’ Performance

Since we compared wolves and dogs at the age of 6 months, the higher success and imitative performance of wolves in contrast to dogs could possibly be explained by the faster cognitive (and physical) development of wolves. In order to verify that not a potential developmental difference between dogs and wolves drove the observed results, we retested 13 of the 15 dogs after a minimum of 9 months. Each dog saw the same demonstration as the first time, that is, they had even a better chance to learn and succeed than the wolves had in the first experiment. Despite of this, again only four dogs were successful. That is, we found the same difference between young wolves and adult dogs as in the first experiment (Fisher exact test: p<0.001; see figure 2).

Interestingly, only one of the 4 successful adult dogs was successful also in the first experiment: Binti had opened the box once in the first experiment and again only opened the box a single time in the repetition task. Two females that were successful during the first round of testing could not open the box in the repetition. However, two males that were previously unsuccessful improved their performance opening the box 4 and 6 times respectively. Finally, like in the first testing, the older dogs also did not match the demonstrated action in their first manipulation (binomial: p = 0.63; 3 same vs. 1 different).

### The Influence of the Demonstration on the Problem Solving Performance of Wolves

We found no difference between the two groups in time they needed to approach the box to within 1 m (exact Mann-Whitney U test: N_T_ = 10, N_C_ = 5, z = −1.65, p = 0.11), no difference between the two groups was found in the latency to contact the lever the first time (exact Mann-Whitney U test: N_T_ = 9, N_C_ = 5, z = −0.47, p = 0.68). Nevertheless, the wolves in the control group started later to manipulate the box than the wolves in the test group (Mann-Whitney U test: N_T_ = 10, N_C_ = 5, z = −2.694, p = 0.005), but the two groups did not differ in regard to which part of the box they manipulated first (Fisher exact test: p<0.99; control: 2 handle vs. 3 other; test: 6 handle vs. 5 other).

Overall, the wolves tested in the control group differed in success from the wolves that saw a full demonstration (Fisher exact test: p = 0.003, see figure 2). All wolves in the test group were successful, while only 1 of the 5 control animals was successful in opening the box by manipulating the lever at least once.

## Discussion

To our knowledge, these data provide the first comparison of social learning and in particular imitation in wolves and equally raised and kept dogs. Our findings show that wolves were more successful and were more likely to copy the actions of conspecifics compared to dogs. Interestingly, we found no significant difference between wolves and dogs in the latency to approach the box, to investigate the lever, which was manipulated during the demonstration by the model, suggesting that in both groups a simple enhancement effect directed the first approach and investigation of the animals towards the same location. However, wolves and dogs seemed to have extracted different details from the demonstrations. While at least half of the wolves started to manipulate the lever of the box first, the majority of the dogs first manipulated another part of the box. In line with this, all wolves succeeded in the first trial and generally continued to open the box, while only 4 dogs were successful in the first trial and only two of those were able to open the box more than once. This difference cannot be attributed to a difference in motivation, since the latency to manipulate the front of the box was the same for wolves and dogs.

One possible explanation for this observed difference is that wolves have a better physical insight as proposed by Frank (1980), and simply succeed better in solving this instrumental problem. Earlier studies by Frank and colleagues found that wolves were more successful than similarly raised dogs in different problem-solving and manipulation tasks at the age of 6- and 10-weeks [34], [35], [36], suggesting a better understanding of rudimentary means-ends relations [8]. This hypothesis would predict that wolves 1) are better problem-solvers than dogs, and 2) can better recognize the connection between pushing the lever down and the opening of the box if they have the chance to observe somebody else doing it or try it out themselves. In contrast with the first prediction, however, we found that in absence of a demonstration the wolves were rather unsuccessful in opening the box as shown by our control group. Whether or not the wolves in the demonstration group benefited from enhanced means-end understanding as assumed by prediction 2 is unclear. More importantly, however, this hypothesis cannot explain why the wolves matched their action to that of the demonstrator instead of focusing purely on pushing the lever down in any way. Finally, two recent studies on physical understanding in wolves and dogs call Franks’ hypothesis into questions: First, testing a larger sample of adult wolves (N = 9 compared to N = 4 in the original studies by Frank and colleagues) in a string-pulling task we found no evidence for wolves being better in recognizing means-end connections than kennel kept sledge dogs [37]. Second, another study investigating object permanence skills found no evidence for better physical understanding in wolves than dogs [38].

Alternatively, it has been proposed that dependency on their owners prevents dogs from solving problem tasks efficiently and independently. It has been proposed that instead of doing so they approach the human participants of problem situations and ask for their help [39], [40]. However, in our experiment the wolves started to manipulate the box significantly earlier than the dogs looked at one of the experimenters for the first time and they even succeeded to open the box by the time the dogs looked at a human. Thus, while the dogs did approach and look at the humans, they only did so after sufficient time had elapsed that allowed the wolves to open the box and would probably have been enough also for them to succeed (since we found no significant difference between wolves and dogs in their latency to manipulate the front of the box). However, alternatively it is still possible that dogs need longer to imitate an action than wolves and that thus, dependency on the human might have influenced their problem solving ability.

Thirdly, one could propose that the failure of the 6-months old dogs in the first experiment of this study is due to their delayed physical or cognitive development compared to wolves [41]. However, when we retested the dogs approximately 9 months later, they were not more successful than at a young age despite having seen again 6 demonstrations. Also, they did not match the demonstrators in their first manipulation on the lever, showing that their imitative skills did not reach that of young wolves even at this adult age. These results have been confirmed by a previous study with adult pet dogs (>1 year of age) using the same experimental apparatus, in which we found that only 8 of 33 dogs successfully opened the box after having observed a dog demonstration with 5 of the 8 dogs being highly trained [34].

To sum up, we found that our wolves could profit more from a conspecific demonstration than our pack-living dogs when solving a manipulative task. The advantage of the wolves presented itself in their higher success, which was likely due to having learnt which part of the box to manipulate in order to access the food as well as matching their actions on the lever to that of the demonstrators. A few studies have demonstrated that dogs are capable of imitating conspecifics in certain situations [26], [35] but the current study, similarly to other findings (e.g. [25], [34]), shows that this skill does not necessarily appear in every situation when imitation would be possible. Rather it seems that the wolves were sensitive towards the details of the action demonstrated by a conspecific compared to the dogs even when we controlled for their overall attentiveness by repeating demonstration trials in which the animals were clearly distracted (see also [42] for a discussion on different levels of social attentiveness). This might reflect a generally higher tendency of wolves to pay close attention to conspecifics, which confirms the predictions of the *canine intraspecific cooperation hypothesis*. Paying attention towards the exact behavior of others is a prerequisite not just for social learning, but also for the coordination of actions and thus might be more important for wolves than dogs that rely less on intraspecific cooperation. In primates, differences in the perceptual and attentional mechanisms underlying information-processing strategies - like relying on global versus local precedence - have been suggested to account for differences in learning [43]. Albeit not all results on social learning in dogs can be explained in this way (see e.g. [30]), a recent study showed that dogs have a preference for global over local cues (global precedence) in the visual processing of geometrical stimuli [44] and potentially also during facial processing [45](but see [46]). Whether wolves, however, have a local precedence is currently unknown, but recent technical developments may allow testing this hypothesis by using eye-tracking equipment also with wolves in the future [47], [48].

Overall, we propose that the canine intraspecific cooperation hypothesis is better suited to make predictions about wolf cognition than the domestication hypotheses, and is likely to present a complementary or alternative way to reason about the evolutionary origins of dog-human cooperation in addition to domestication. According to this hypothesis, dog-human cooperation has likely originated from wolf-wolf cooperation, potentially by dogs’ becoming able to easily accept humans as social partners and thus, extending their relevant social skills to interactions with them [3], [6].

In human and non-human primates, recent results suggest a close bidirectional link between cooperativeness and ‘simple’ imitation (or mimicry) [49], [50]. For example, it has been shown that humans and capuchins who have been imitated by an interaction partner are more altruistic [51] or, in the case of the monkeys, interact more frequently with imitators in a token exchange task [52]. Vice-versa, prosocial attitudes lead to increased mimicry in humans [53]. Two different effects of social attitudes on mimicry have been proposed: direct effects, where social attitudes increase the probability of copying, and indirect effects, where the amount of attention given by the observer to the body movements of the model is modulated. Lakin and Chartrand [54] argued that if people have the urge to affiliate it might cause them to pay closer attention to what occurs in their social environments (i.e. they perceive more). These arguments might not just be relevant for mimicry, but by increasing attention towards others it allows for close coordination as well as social learning to become possible [55]. Accordingly the close social cohesion of cooperative species may lead to higher attention towards their social environment.

Overall, this study is the first to demonstrate that wolves, a non-primate species characterized by cooperative breeding, is capable of imitation, and thus, tests in a broader comparative perspective the hypothesis that cooperative animals are likely to pay closer attention to the actions of social partners and thus may have a higher tendency to socially learn from or even imitate each other’s actions.

## Supporting Information

Video S1 **Paw demonstration for a dog subject.**
(M4V)

Video S2 **Paw demonstration for a wolf subject.**
(M4V)

Video S3 **Dog trying to solve the task in the test phase after observing a paw demonstration.**
(M4V)

Video S4 **Wolf trying to solve the task in the test phase after observing a paw demonstration.**
(M4V)
